# Silencing of *DND1* in potato and tomato impedes conidial germination, attachment and hyphal growth of *Botrytis cinerea*

**DOI:** 10.1186/s12870-017-1184-2

**Published:** 2017-12-06

**Authors:** Kaile Sun, Ageeth van Tuinen, Jan A. L. van Kan, Anne-Marie A. Wolters, Evert Jacobsen, Richard G. F. Visser, Yuling Bai

**Affiliations:** 1 Plant Breeding, Wageningen University & Research, Droevendaalsesteeg 1, 6708 PB Wageningen, The Netherlands; 2 Laboratory of Phytopathology, Wageningen University & Research, Droevendaalsesteeg 1, 6708 PB Wageningen, The Netherlands

**Keywords:** *Botrytis cinerea*, *DND1*, Infection progress, Necrotroph, Plant disease, Susceptibility gene

## Abstract

**Background:**

*Botrytis cinerea*, a necrotrophic pathogenic fungus, attacks many crops including potato and tomato. Major genes for complete resistance to *B. cinerea* are not known in plants, but a few quantitative trait loci have been described in tomato. Loss of function of particular susceptibility (*S*) genes appears to provide a new source of resistance to *B. cinerea* in Arabidopsis.

**Results:**

In this study, orthologs of Arabidopsis *S* genes (*DND1, DMR6*, *DMR1* and *PMR4*) were silenced by RNAi in potato and tomato (only for *DND1*)*. DND1* well-silenced potato and tomato plants showed significantly reduced diameters of *B. cinerea* lesions as compared to control plants, at all-time points analysed. Reduced lesion diameter was also observed on leaves of *DMR6* silenced potato plants but only at 3 days post inoculation (dpi). The *DMR1* and *PMR4* silenced potato transformants were as susceptible as the control cv Desiree. Microscopic analysis was performed to observe *B. cinerea* infection progress in *DND1* well*-*silenced potato and tomato leaves. A significantly lower number of *B. cinerea* conidia remained attached to the leaf surface of *DND1* well-silenced potato and tomato plants and the hyphal growth of germlings was hampered.

**Conclusions:**

This is the first report of a cytological investigation of *Botrytis* development on *DND1*-silenced crop plants. Silencing of *DND1* led to reduced susceptibility to *Botrytis,* which was associated with impediment of conidial germination and attachment as well as hyphal growth. Our results provide new insights regarding the use of *S* genes in resistance breeding.

**Electronic supplementary material:**

The online version of this article (10.1186/s12870-017-1184-2) contains supplementary material, which is available to authorized users.

## Background

*Botrytis cinerea*, a necrotrophic pathogen which causes grey mold disease, can infect a broad range of plant species including potato and tomato. Every year, global expenses of *Botrytis* control surmount €1 billion [1]. Hence, *B. cinerea* has become the most extensively studied necrotrophic fungal pathogen. Necrotrophic pathogens damage plant cells by secreting toxic compounds, lytic enzymes and an array of pathogenicity factors which can subdue host defences [2]. In order to penetrate the plant cuticle and epidermal cells, *B. cinerea* produces cutin esterases, pectinases and hemicellulases during the early infection stages, between 8 and 16 h post inoculation (hpi) [3–6]. At later stages, between 16 and 40 hpi, multiple phytotoxic metabolites such as botrydial, botcinic acid and oxalic acid, as well as several phytotoxic proteins are produced and secreted into epidermal and mesophyll tissue to kill the host cells [2, 6].

The infection process in susceptible potato and tomato leaf tissue starts when conidia land on the leaf. Prior to germination, conidia adhere weakly to the plant surface by weak hydrophobic interactions [7, 8]. When inoculated in nutrient solution, conidia generally germinate within 3–4 h and form one or two germ tubes that can elongate to 20–40 μm, and are covered with a polysaccharide glucan matrix [9, 10] that increases the adhesion to the plant surface. Around 6 hpi, the tip of the germ tube develops a swollen, non-melanized appressorium [11] that is tightly attached to the plant surface by a mucilage. In the absence of melanin, the appressorium does not exert high physical pressure, but it uses hydrolytic enzymes to penetrate the plant cuticle around 8 hpi. Fungal hyphae subsequently grow inside host tissue over a limited distance, with no visible signs of host cell death until ~14–16 hpi. The infection zone may at this time point develop water-soaked pinpoint lesions that result from degradation of host cell walls, however, the infection droplet is colourless. From 16 to 18 hpi onwards, plant cells begin to collapse and turn necrotic, and the infection droplet turns in colour by the accumulation of brown (phenolic) compounds [12]. Typical scanning electron microscopy (SEM) images of the different stages of infection on broad bean are provided in Tenberge (2007) [13].

No major genes (*R* genes) for complete resistance to *B. cinerea* have been identified in plants. However, quantitative trait loci (QTLs) reducing the outgrowth of *B. cinerea* have been reported in several plant species, first in Arabidopsis [14]. In the wild tomato accession LYC4 from *Solanum habrochaites*, ten major QTLs have been described for reduced lesion growth and for reduced disease incidence [15]. More recently, QTLs have been identified for resistance to *B. cinerea* in chickpea and *Brassica rapa* [16, 17].

Recently, plant genes (susceptibility or *S* genes) that are recruited by pathogens to promote diseases have become the focal point of research attention [18]. In Arabidopsis and tomato, 16 plant genes have been reported that participate in disease development by *B. cinerea* (Additional file 1: Table S1)*.* Examples of these are the tomato genes, *LePG* and *LeEXP1*, which contribute to fruit cell wall softening during ripening and thereby facilitate penetration and colonization by *B. cinerea* [19], as well as the Arabidopsis *CESA4, CESA7* and *CESA8* genes, encoding subunits of the cellulose synthase complex [20].

Evidence is accumulating that loss-of-function mutations of certain plant *S* genes can result in resistance to pathogens [21–25]. In tomato, the ABA deficient mutant *sitiens* is highly resistant to *B. cinerea* [26–28]. In Arabidopsis, the invasion of *B. cinerea* was arrested in the *bre1* mutant [29]. The *BRE1* gene, also known as the *long-chain acyl-CoA synthetase2* (*LACS2*) gene, is essential for cutin biosynthesis. Furthermore, the Arabidopsis *dnd1* (*defense no death*) mutant showed enhanced resistance against a range of fungal, bacterial and viral pathogens, including *B. cinerea* [30–32]. In our previous study, we reported that silencing of potato orthologs to *DND1*, *DMR6*, *DMR1* and *PMR4* resulted in resistance to late blight [33]. These experiments demonstrated that plant *S* genes are conserved across plant species with respect to their function as a susceptibility factor to certain types of pathogens.

In this study, we aimed to test whether impairing the functionality of *S* genes in potato would confer resistance to *B. cinerea*. Potato RNAi transformants, in which orthologs of *DND1, DMR6*, *DMR1* and *PMR4* were silenced, were examined for reduced susceptibility to *B. cinerea*. In addition, we extended the analysis to *DND1*-silenced tomato plants and performed microscopy to follow the development of *B. cinerea* germlings on leaf surfaces.

## Results

### *StDND1* well*-*silenced potato plants show reduced susceptibility to *Botrytis cinerea*

**Fig. 1:**
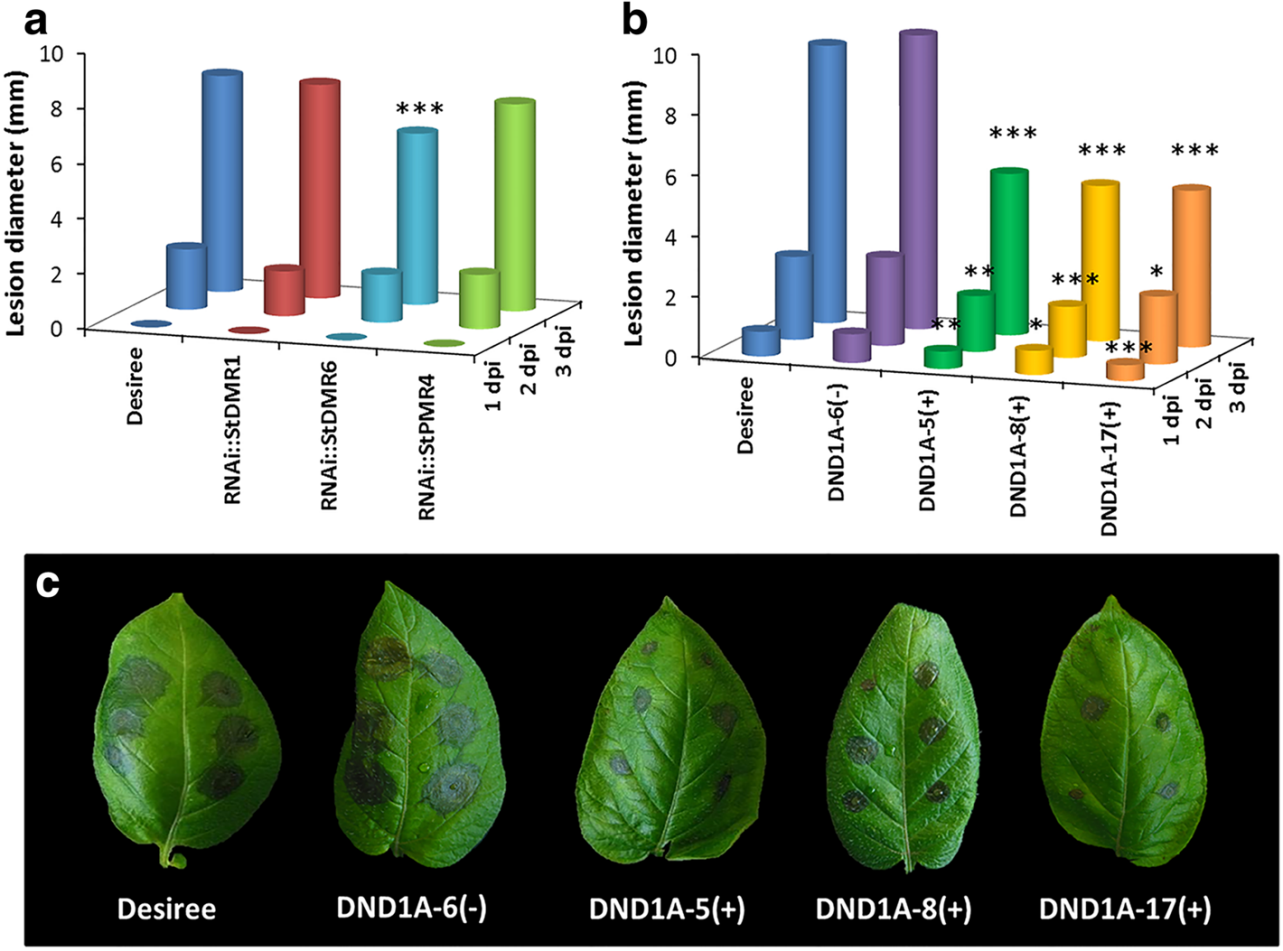
Detached leaf assay (DLA) of potato RNAi transformants with *Botrytis cinerea* (strain B05.10). **a** and **b** Lesion diameter on the inoculated leaves of the RNAi transformants. **a** Results from one of the two independent experiments performed for *StDMR1*, *StDMR6* and *StPMR4* (two independent well-silenced transformants per gene were used in each experiment); **b** Results from one of the three independent experiments for *StDND1.* Four independent transformants were used, one weakly-silenced transformant (−), and three well-silenced transformants (+). Susceptible control was cv Desiree. The average of all lesion diameters per transformant per time point is provided. Asterisks indicate degree of significance compared to cv Desiree plants (*p < 0.05, **p < 0.01, ***p < 0.001); **c** *B. cinerea* disease symptoms on cv Desiree and *StDND1*-silenced transformants. Photos were taken at 3 days post inoculation (dpi)

In potato, silencing of the genes *DND1*, *DMR1*, *DMR6* and *PMR4* resulted in reduced susceptibility to late blight. In order to assess whether silencing these *S* gene candidates also leads to reduced susceptibility to *B. cinerea,* RNAi well-silenced transformants of *StDMR1*, *StDMR6, StDND1* and *StPMR4* were inoculated with the wild type strain B05.10 in a detached leaf assay (DLA). In all these transformants, except DND1A-6, the expression level of the corresponding target gene is significantly down-regulated (> 60% reduced *S* gene expression [33]). The leaves of cv Desiree were used as susceptible control. Lesion diameters on infected leaves were measured daily from 1 to 3 days post inoculation (dpi) (Fig. 1). Desiree and the DND1A-6(−) (RNAi weak-silenced transformant) showed similar *B. cinerea* lesions (Fig. 1). Compared to cv Desiree, significantly smaller *B. cinerea* lesions were observed on *StDMR6* RNAi well-silenced transformants at 3 dpi and on *StDND1* RNAi well-silenced transformants at all three time points (Fig. 1, Additional file 2: Figure S1 and Additional file 3: Figure S2).

### *B. cinerea* conidial surface attachment and germling growth are reduced on leaves of *StDND1* well*-*silenced potato plants

**Fig. 2:**
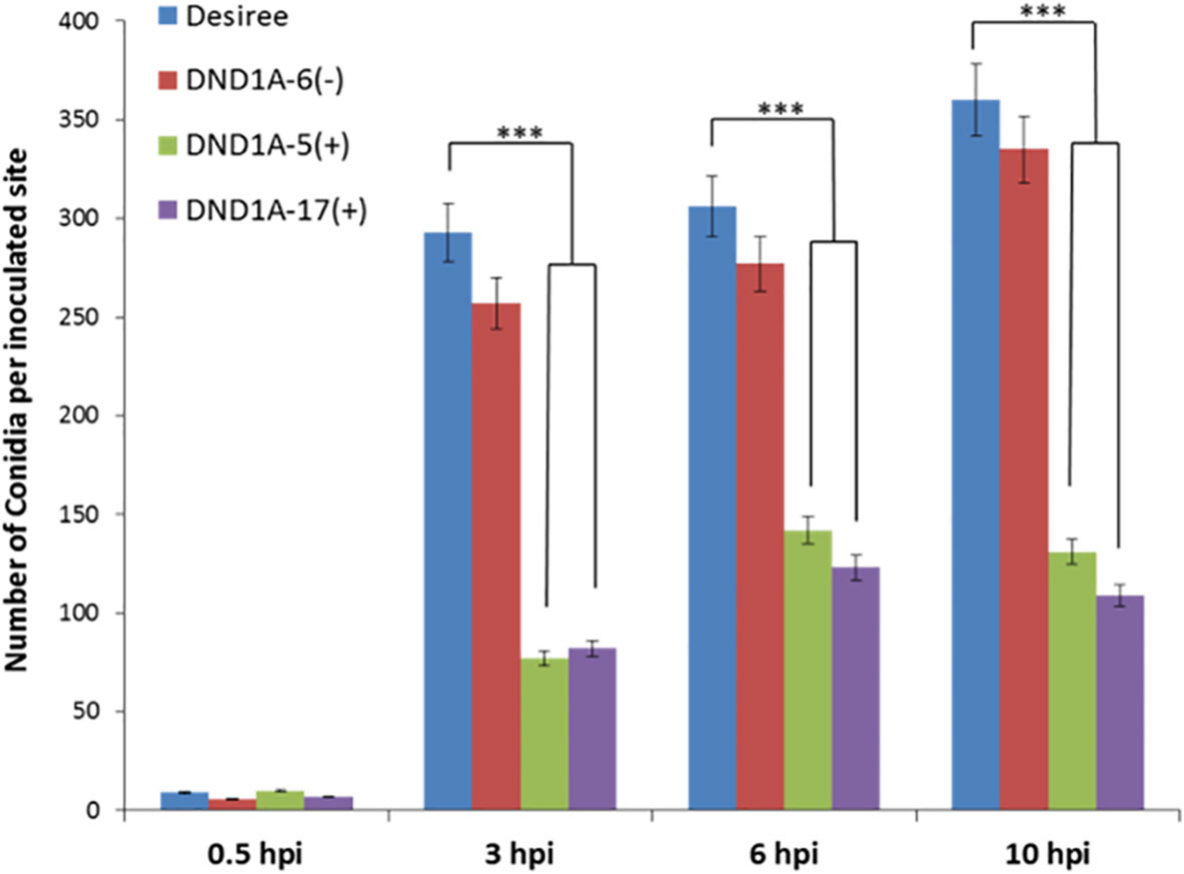
Number of *Botrytis cinerea* (strain SAS56 containing pCutGUS) conidia on infected leaves of cv Desiree and *StDND1*-silenced potato plants. From cv Desiree and each transformant, three plants were tested (one leaf with 5 leaflets per plant). Bars indicate means ± standard deviation. Asterisks indicate degree of significance compared to cv Desiree plants (***p < 0.001)

**Fig. 3:**
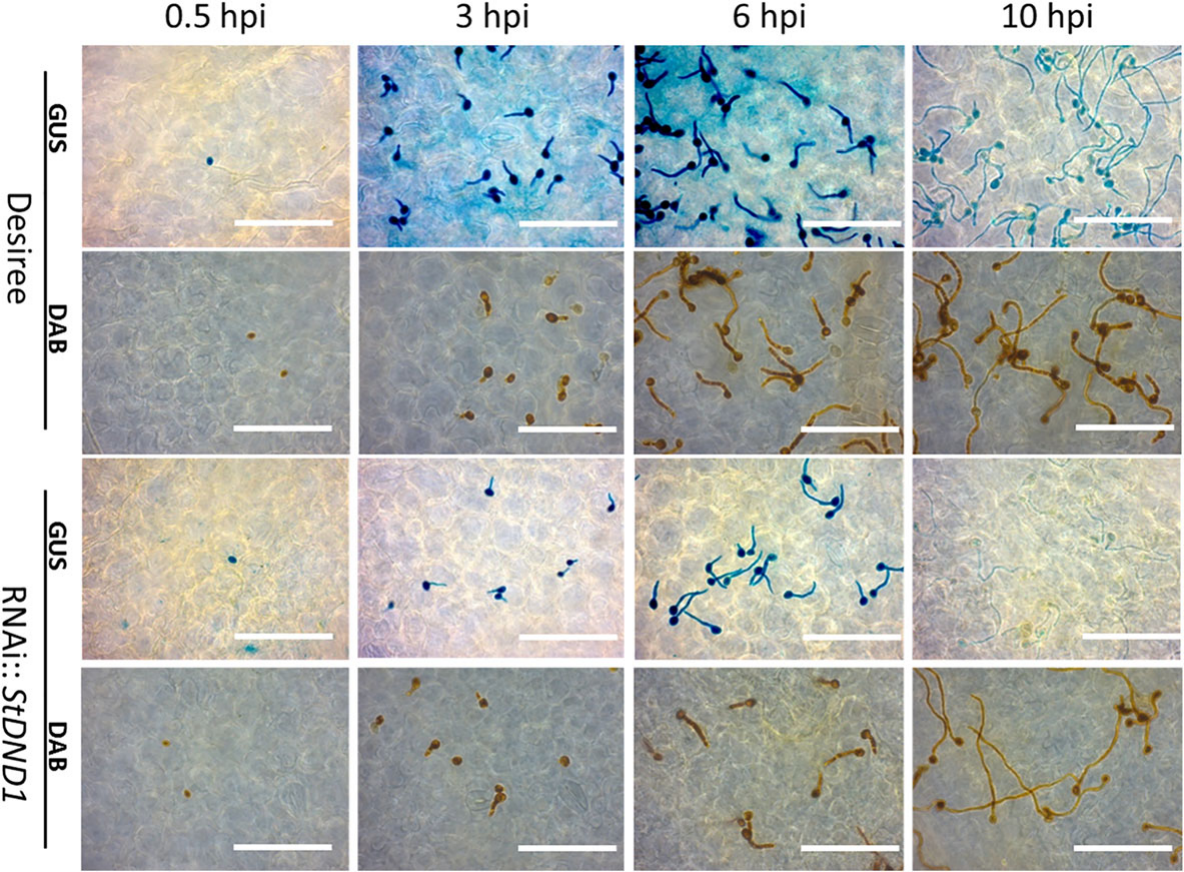
Microscopic observations after GUS and DAB staining of *Botrytis cinerea* inoculated cv Desiree and *DND1* well-silenced potato plants (Scale bar = 100 μm). The same plants were used in the conidia counting in Fig. 2. Samples were taken for GUS and DAB staining at 0.5, 3, 6, and 10 h post inoculation (hpi), respectively. From each genotype, three plants were tested (5 leaflets per plant) and four slides per time point per plant were observed. The photos are representative for the results observed in twelve preparations per genotype

In order to get insight into the mechanism of reduced susceptibility, a histological study was performed on the fungal spore germination and outgrowth of germlings in *B. cinerea*-infected leaves of *StDND1*-silenced potato plants and of cv Desiree. A *B. cinerea* reporter strain was used that contains a GUS gene under control of the promoter of the cutinase gene *Bccut*A, which is inactive in spores but induced from the onset of germination onwards [34]. GUS activity and accumulation of H_2_O_2_ were monitored microscopically following histochemical staining and the number of spores per inoculation droplet was counted (Fig. 2). At 0.5 hpi, only few blue stained conidia could be observed on the leaf surfaces of all genotypes, partly because the *BccutA* promoter was at that moment inactive [34] and partly because conidia were washed from the leaf surface in the staining procedure due to their weak attachment to the cuticle [7]. At 3 hpi, the majority of conidia had germinated on cv Desiree and the germ tubes increased in length at subsequent time points, 6 and 10 hpi. Both conidia and germ tubes stained blue due to the induction of the *Bccut*A promoter during infection (Fig. 3). The total number of blue-stained conidia on the leaf surfaces at the three time points tested (3, 6, and 10 hpi) did not significantly differ between cv Desiree and the weakly-silenced plant DND1A-6 (Fig. 2). However, on the leaves of two *StDND1* well-silenced plants the number of blue-stained conidia was two- to three-fold lower than on cv Desiree at all-time points analysed (Fig. 2). Fig. 3 shows that the blue GUS stain of fungal hyphae decreased to some extent at 10 hpi on cv Desiree, but vanished almost entirely in the *StDND1* well*-*silenced potato plants (see also Additional file 4: Figure S3). DAB staining showed that H_2_O_2_ accumulated on the surface of fungal conidia and germ tubes (Fig. 3), as a result of the activity of glucose oxidase and superoxide dismutase, enzymes that are produced by *B. cinerea* and excreted to the fungus-host interface [35, 36]. The generation of H_2_O_2_ on the fungal matrix [37] is not harmful to *B. cinerea* and the fungus does not appear to experience intracellular oxidative stress during infection [38]. In most cases, there was a correspondence between the GUS staining and DAB staining in fungal tissue, with one exception. Notably, at 10 hpi on *StDND1* well*-*silenced potato plants, DAB staining detected clear accumulation of H_2_O_2_ on the fungal germ tube surfaces, even though fungal structures were barely detected following the GUS staining (Fig. 3). This observation suggested that fungal hyphae were damaged, possibly to such an extent that cytoplasmic content leaked out and GUS activity vanished from the hyphae, while H_2_O_2_ generating enzymes on the hyphal surface remained active.

**Fig. 4:**
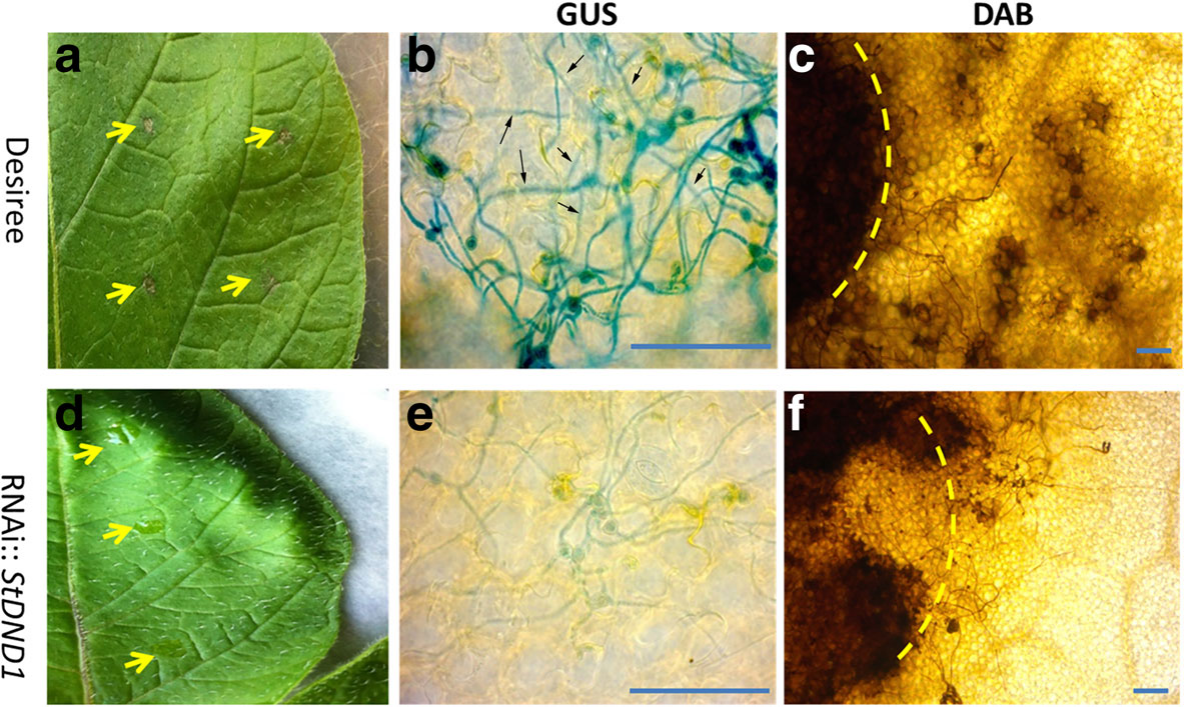
Disease symptoms of *Botrytis cinerea* (strain SAS56 containing pCutGUS) on inoculated leaves of cv Desiree and *StDND1* well-silenced potato plants at 24 h post inoculation (Scale bar = 100 μm). **a** and **d** photos of *B. cinerea* infected leaves of cv Desiree and *StDND1* well-silenced potato plants. Yellow arrows point to the inoculation sites, where lesions can be seen on the cv Desiree leaf but not on the *StDND1* well*-*silenced leaf. **b** and **e** and **c** and **f** GUS and DAB staining of leaf samples from *B. cinerea* infected cv Desiree and *StDND1* well-silenced potato plants. The small black arrows in **b** mark hyphae extending below the plane of focus

At 24 hpi, necrotic lesions could be observed on the inoculation sites of cv Desiree leaves (Fig. 4a) which had the size of the initial inoculation droplet. Hyphae of *B. cinerea* could be observed that were extending below the plane of focus, indicating that hyphae might be underneath the leaf cuticle (Fig. 4b). On the leaves of *StDND1* well*-*silenced plants, necrotic lesions were not visible (Fig. 4d) and only a small number of *B. cinerea* hyphae were observed that appeared to be growing mostly on the leaf surface and displayed very weak GUS staining (Fig. 4e). Accumulation of H_2_O_2_, as shown by dark DAB staining, was observed beyond the inoculation sites on cv Desiree plants (Fig. 4c). In contrast, DAB staining was limited to the inoculation sites on the *StDND1* well*-*silenced plants and the intensity was less pronounced (Fig. 4f).

**Fig. 5:**
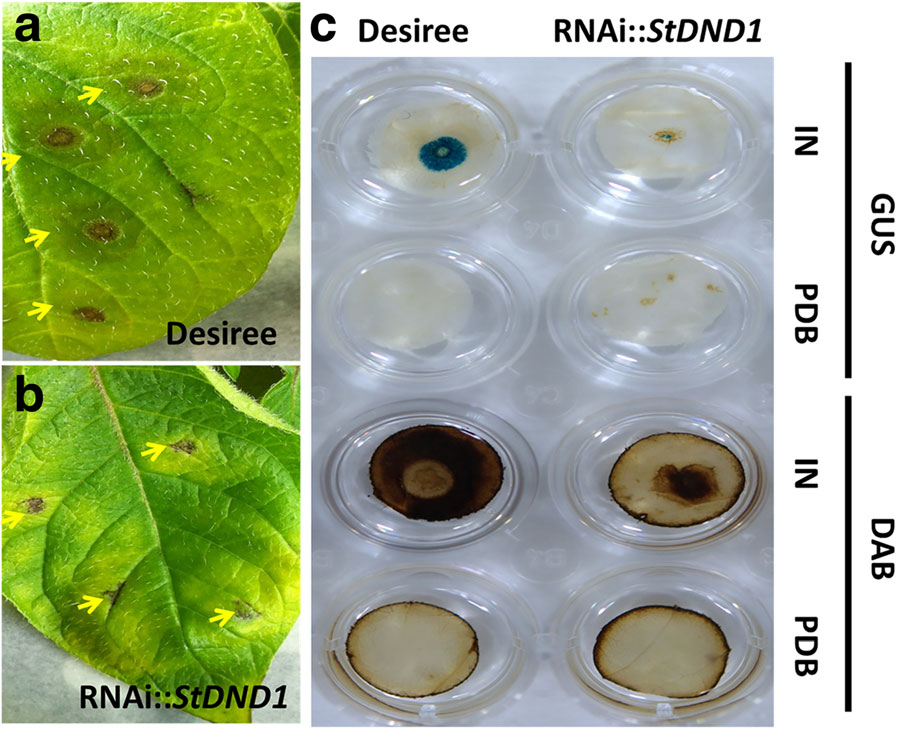
Disease symptoms of *Botrytis cinerea* (stain SAS56 containing pCutGUS) on inoculated leaves of cv Desiree and *StDND1* well-silenced potato plants at 48 h post inoculation. Left panel (**a** and **b**) photos of *B. cinerea* infected leaves of cv Desiree and *StDND1* well-silenced plants. Yellow arrows point to necrotic spots occurring at the inoculation sites. Right panel (**c**) GUS and DAB staining of leaf samples with inoculation sites from cv Desiree and *StDND1* well*-*silenced potato plants after *B. cinerea* inoculation (IN) or mock treatment (PDB). GUS stains hyphae of *B. cinerea* as blue spots at inoculation sites. DAB stains the H_2_O_2_ accumulation as brownish areas at inoculation sites. IN, inoculated; PDB, Potato Dextrose Broth medium; GUS, beta-glucuronidase; DAB, 3,3′-diaminobenzidine

At 48 hpi, lesion development of *B. cinerea* was observed by naked eye on both cv Desiree and *StDND1* well-silenced potato plants (Fig. 5, a and b). Necrotic spots, surrounded by a water-soaked lesion, developed on inoculated leaves of cv Desiree. Much smaller necrotic spots were observed on the *StDND1* well-silenced potato plants and they were surrounded by chlorotic tissue rather than a water-soaked area typically observed in the control. Leaf samples of the inoculation sites were taken from both cv Desiree and *StDND1* well-silenced potato plants after inoculation with *B. cinerea* or after mock-treatment (Potato Dextrose Broth, PDB) and were stained with GUS and DAB (Fig. 5c). GUS staining resulted in deep blue spots on leaves of cv Desiree, reflecting a dense growth of *B. cinerea* hyphae inside the necrotic lesion, but not in the water-soaked area. Leaves of *StDND1* well-silenced potato plants exhibited much smaller blue spots, surrounded by a yellow/brownish ring, indicating a restricted extent of fungal growth surrounded by plant tissue undergoing chlorosis and cell death (yellow/brownish colour). No fungal growth was observed on leaves mock-inoculated with only PDB (Fig. 5). DAB staining showed that the brown colour in the wild type (cv Desiree) control extended well beyond the borders of the lesion, where the fungus could not be detected by GUS staining, suggesting that an oxidative burst occurred in plant tissue at a significant distance from the front of the expanding fungal hyphae. In contrast, the inside of the lesion lost most of its brown colour, as the plant tissue was entirely colonized by the fungus and no longer capable of mounting an oxidative response to the presence of the fungus. In *StDND1* well*-*silenced potato plants, however, the brown colour seemed to be restricted to the necrotic zone surrounding the fungal hyphae, and the entire brown region was smaller than in the control. Moreover, the central part of the inoculation site in *StDND1* well*-*silenced plants was deep brown and not discoloured like in the control (Fig. 5), suggesting a pronounced oxidative burst in the plant tissue in the vicinity of the invading fungal hyphae.

### Expression of *B. cinerea* endopolygalacturonase gene *Bcpg*1 and cutinase gene *BccutA* are altered in *StDND1* well-silenced potato

**Fig. 6:**
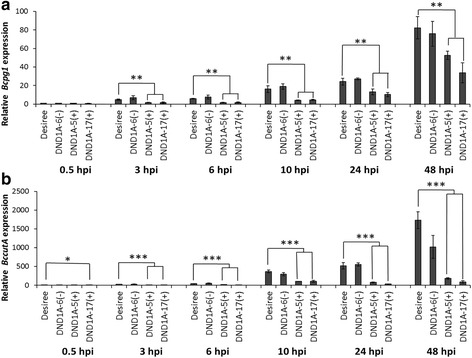
Relative transcript levels of the *Botrytis* polygalacturonase gene *Bcpg1* and the cutinase gene *BccutA* in cv Desiree and *StDND1*-silenced potato transformants during infection with *B. cinerea* (strain SAS56 with a pCutGUS). Infected leaves were sampled at 0.5, 3, 6, 10, 24, and 48 h post-inoculation (hpi) for RNA extraction. For each sample, the transcript level of *Botrytis* gene *Actin* was used as reference, the relative expression level of the target gene in cv Desiree leaves at time point 0.5 hpi was defined as 1. Data represent the mean of three biological replicates with error bars displaying the standard error. Three technical replicates of each repeat were analysed, which showed similar results. Asterisks indicate degree of significance compared to cv Desiree plants (*p < 0.05, **p < 0.01, ***p < 0.001)

For *B. cinerea*, successful penetration of the cell wall requires enzymes capable of degrading pectin, including endopolygalacturonases (endo-PGs) and pectin methylesterases (PMEs). In order to assess whether the expression of one of the endo-PG genes was affected in *StDND1* well*-*silenced potato plants during infection, the transcript levels of *Bcpg1* were investigated from 0.5 to 48 hpi (Fig. 6a). The expression level of *Bcpg1* in leaves of cv Desiree and the *DND1* weak-silenced potato DND1A-6(−) increased 5–10 fold at 3 hpi and continued to rise until the last time point at 48 hpi. The transcript levels of *Bcpg1* in the two *StDND1* well*-*silenced potato transformants DND1A-5(+) and DND1A-17(+) only started to increase somewhat at 10 hpi, while at 24 hpi they reached values that were comparable to the 6 hpi sample on the control plants. Only at 48 hpi did the *Bcpg*1 transcript levels in *StDND1* well*-*silenced potato plants approach the level in both cv Desiree and potato transformant DND1A-6(−), but the difference in transcript level was still significant.

To assess whether the expression of the *B. cinerea* cutinase gene was altered in *DND1* well-silenced potato plants, the transcript levels of *BccutA* were also investigated in cv Desiree and *StDND1*-silenced potato transformants from 0.5 to 48 hpi (Fig. 6b). Similar to the transcript pattern of *Bcpg1*, significantly lower transcript levels of *BccutA* were observed in *StDND1* well*-*silenced potato transformants. These results are in agreement with GUS staining (Fig. 3) indicating that the growth of *B. cinerea* is reduced in *StDND1* well*-*silenced potato plants.

### *SlDND1* well*-*silenced tomato plants show reduced susceptibility to *Botrytis cinerea*

**Fig. 7:**
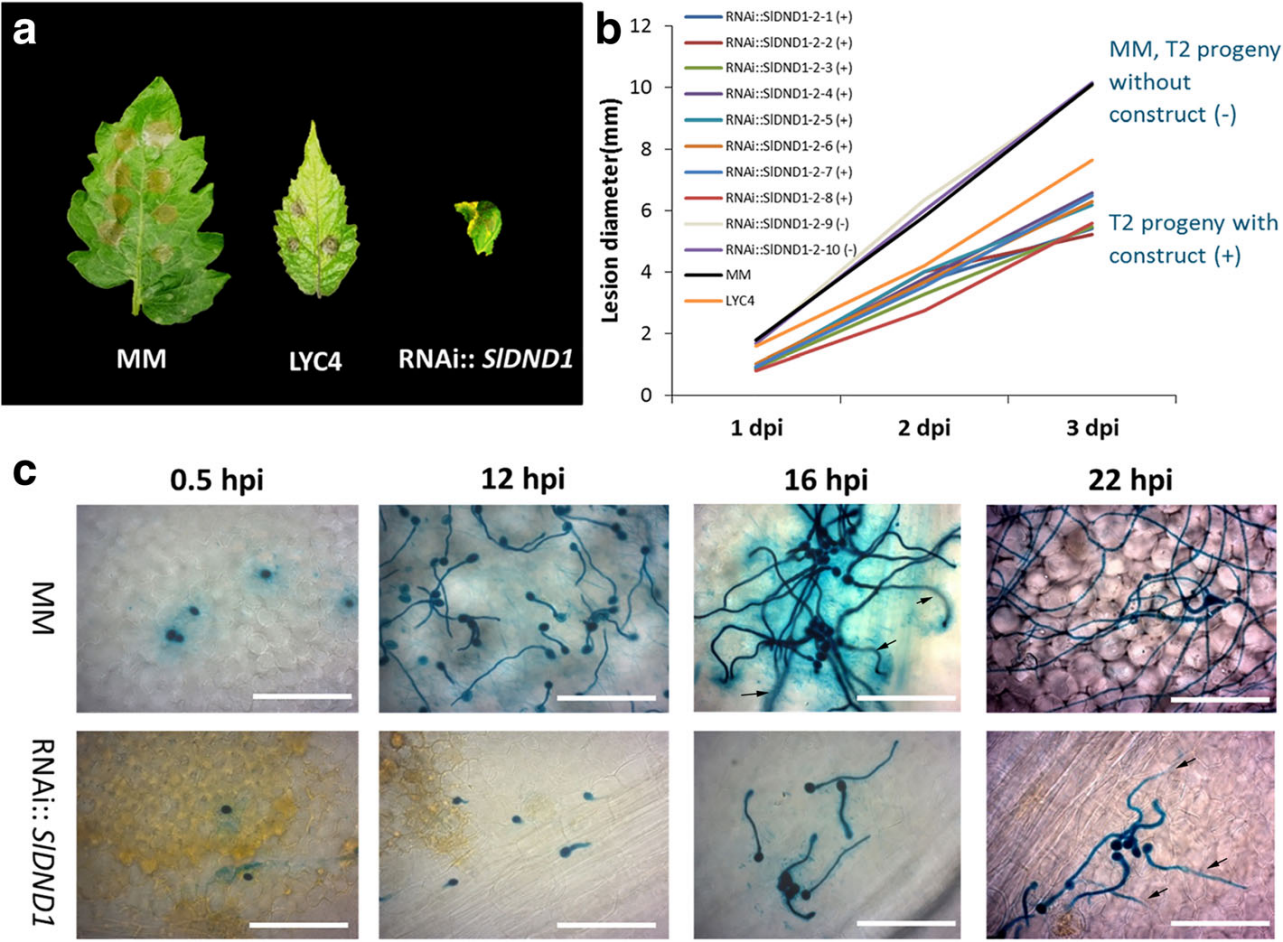
Infection progress of *Botrytis cinerea* on *SlDND1-*silenced tomato transformants at different time points after inoculation*.* **a** Disease symptoms of *B. cinerea* on leaves of cv Moneymaker (MM), *S. habrochaites* genotype LYC4 and *SlDND1-*silenced tomato plants. **b** Lesion development in T2 progeny (T2#2 family derived from T1#2, Sun et al. 2016) harbouring a *SlDND1* silencing construct (+, *n* = 8) or their non-transgenic siblings (−, *n* = 2)*.* Data were collected at 1, 2, and 3 days post inoculation (dpi)*.* For each genotype, five leaflets per plant were inoculated with *B. cinerea* (strain B05.10). **c** Infection progress of *B. cinerea* (strain SAS56 containing pCutGUS) on MM and *SlDND1* well*-*silenced tomato plants at four time points after inoculation (Scale bar = 100 μm). The small black arrows mark hyphae extending below the plane of focus (at 16 hpi in MM and 22 hpi in RNAi *DND1* silenced plants). The photos were taken at 0.5, 12, 16, and 22 h post inoculation (hpi), respectively. The photos are representative for the results observed in six preparations per genotype

In order to investigate whether silencing *DND1* can reduce *B. cinerea* infection also in tomato, the second generation (T2) of RNAi::*SlDND1* tomato plants obtained after transformation of cv Moneymaker (MM) [23] were used in a DLA with *B. cinerea* strain B05.10. T2 plants were screened for the presence or absence of the RNAi construct. Compared with the susceptible control MM, similar sizes of necrotic spots were found on non-silenced tomato T2 plants (not carrying the RNAi construct). In contrast, smaller necrotic spots were observed at the inoculation sites on the *SlDND1* well*-*silenced tomato T2 plants (carrying the RNAi construct) and the partially resistant control genotype, *Solanum habrochaites* accession LYC4 (Fig. 7a). From 1 to 3 dpi, MM and non-silenced T2 plants showed a similar pattern of lesion development (Fig. 7b). The lesion development on *DND1* well-silenced tomato plants was slower than that observed on the partially resistant control LYC4**.** In order to monitor the conidial adhesion, germination and hyphal outgrowth, we performed a histological study with the *B. cinerea* reporter strain containing a GUS gene under control of the promoter of *Bccut*A, as discussed above for potato. Compared to the susceptible MM plants, a lower number of conidia, delayed germination and reduced germ tube growth of *B. cinerea* were observed at 12 hpi on *SlDND1* well*-*silenced tomato plants (Fig. 7c). At 16 hpi, *B. cinerea* hyphae were branching and forming multiple penetration sites on MM plants. At the same time point, on inoculated leaves of *SlDND1* well*-*silenced tomato plants, the growth of hyphae was severely delayed and they were just in the stage of producing appressoria, visible as swellings at the tips of germ tubes. At 22 hpi, *B. cinerea* further developed expanding necrotic lesions on MM plants, but not on *SlDND1* well*-*silenced tomato plants.

## Discussion

In this study four candidate *S* genes, *DND1*, *PMR4*, *DMR6* and *DMR1*, of which the silencing was previously shown to provide resistance to *P. infestans*, were investigated via RNAi in potato for their role in susceptibility to *B. cinerea*. Of these four genes, only *DND1* was tested earlier in relation to *B. cinerea,* and the *dnd1* mutant of Arabidopsis showed increased resistance [30–32]. Here, it turned out that silencing the *DND1* ortholog in potato provided a high level of resistance to *B. cinerea*. Furthermore, silencing the tomato *DND1* ortholog in cv Moneymaker also resulted in reduced susceptibility of tomato to *B. cinerea.* Based on the microscopic observations of the infection process of *B. cinerea* on *DND1* well*-*silenced tomato and potato plants, we discuss here the potential mechanisms by which the silencing of the *DND1* gene confers reduced susceptibility to *B. cinerea*.

The *DND1* gene encodes a protein that belongs to the family of cyclic nucleotide-gated ion channel proteins (CNGCs; [39]. CNGCs have a role in conducting Ca^2+^ into plant cells and are involved in various physiological processes [40]. In the Arabidopsis *dnd1* mutant AtCNGC2 is disrupted. This mutant exhibits a broad-spectrum resistance in absence of hypersensitive response (HR) to several biotrophic and necrotrophic pathogens including *B. cinerea* [41, 42]. HR is one of the most effective ways to impede growth of biotrophic pathogens, however it is considered to facilitate the growth of necrotrophic pathogens like *B. cinerea* [41]. Thus, the resistance of the *dnd1* mutant to *B. cinerea* may be logically explained by the cell death suppression. However, in spite of a deficient HR, a *dnd1ein2* double mutant exhibited susceptibility to *B. cinerea* [30], showing that the ethylene pathway also plays an important role in the elevated resistance to *B. cinerea*.

Our results showed that silencing the *DND1* ortholog in both potato and tomato led to reduced susceptibility to *B. cinerea*. The number of attached *B. cinerea* spores to the plant surface was significantly lower on *DND1* well*-*silenced potato and tomato plants than on wild type plants. In literature, two steps of conidial attachment were described for *B. cinerea*. The first is the weak adhesion to the plant surface via conidia and germ tubes (between 0 to 6 hpi) and the second is a stronger adhesion through appressoria (between 6 to 10 hpi). In our study, similar numbers of blue-stained conidia were found on all potato genotypes at 0.5 hpi (Fig. 2), suggesting germination rate is comparable among these genotypes. At 3, 6 and 10 hpi, significantly lower numbers of conidia was observed on *DND1* well-silenced potato plants, suggesting the adhesion or attachment was impaired. However, in tomato, conidial germination was delayed on RNAi silenced plants compared to that on the control MM plants. Although this is not the case in potato, we cannot rule out the possibility that the conidial germination might be impaired on *DND1* silenced potato plants. Further experiments, such as using *B. cinerea* strains expressing fluorescent proteins and stains like WAG Alexa 488, are helpful to monitor conidial germination, attachment and hyphal penetration on *DND1* silenced tomato and potato plants.

Although it is uncertain, our data may indicate that cuticles of *DND1* well*-*silenced potato and tomato plants are chemically or physically altered. The cuticle consists of a complex polymeric network of esterified hydroxylated fatty acids covered with a wax layer, and it serves, amongst others, as a structural barrier that protects epidermal cells against pathogens [43]. Several cutin-defective Arabidopsis mutants have been identified showing resistance to *B. cinerea*, including *sma4* (also known as *bre1* or *lacs2*), *bodyguard* (*bdg*) and *lacerate (lcr)* (Additional file 1: Table S1). Spore germination and hyphal elongation of *B. cinerea* were inhibited on the leaves of the *sma4* mutant [44]. All these mutants are defective in cuticle integrity and have an increased cuticular permeability. The latter may allow the diffusion of toxic compounds with growth-inhibiting activities, as shown in the *bdg* and *lacs2* mutants [29, 45]. In our study, GUS staining of *B. cinerea* indicated that at 10 hpi, the hyphae appeared to be dead on the *DND1* well-silenced potato plants (Fig. 2). This would be in favour of the hypothesis of diffusion of fungitoxic compounds through a defective cuticle.

There is evidence that cuticular permeability can be uncoupled from resistance to *B. cinerea*. For example, the Arabidopsis *Resurrection 1* (*rst1*) mutant exhibits normal cuticular permeability although it has an altered cuticle composition [46]. This mutant shows resistance to *B. cinerea*, which is associated with attenuated SA-dependent responses and enhanced JA-dependent defence. This resistance was compromised by defective JA and ethylene signalling pathways.

In addition, plant cell walls have a surveillance system. Alteration of cell wall integrity can lead to specific activation of novel defence pathways as in the Arabidopsis *myb46* mutant [47]. The transcription factor MYB46, a key player in regulating secondary cell wall biosynthesis, functions as a susceptibility gene to *B. cinerea* by downregulation of *CesA* genes [48].

We cannot rule out the possibility that the SA signalling pathway plays a role in the weakened *B. cinerea* infection on leaves of *DND1* well*-*silenced potato and tomato plants. In *StDND1* well*-*silenced potato plants, constitutively elevated *PR1* expression was observed [23]. In Arabidopsis, it has been shown that both JA- and SA-mediated signalling are required for local resistance to *B. cinerea* [49]. The Arabidopsis *dnd1* mutant displays elevated SA levels, which could be required for its resistance to a broad range of pathogens including *B. cinerea*. It is, therefore, worthwhile to investigate relative expression of SA, ET and JA pathway marker genes in *DND1* well*-*silenced potato and tomato plants in order to understand the impact of *DND1* silencing on the pathogen’s infection process.

In tomato, resistance to *B. cinerea* is observed in the ABA-deficient *sitiens* mutant by a timely hyper induction of H_2_O_2_-dependent defences in the epidermal cell wall [28]. Further, it was suggested that ABA determines susceptibility to *B. cinerea* by negatively regulating SA signalling [26]. In the tomato *sitiens* mutant, H_2_O_2_ accumulation was observed from 4 hpi in epidermal cell walls in close contact to the fungal germ tubes [28]. In our study, H_2_O_2_ accumulation was associated with the lesion growth in the susceptible control cv Desiree, while in *DND1* well-silenced potato plants, lesions were surrounded by a ring of chlorotic tissue where H_2_O_2_ accumulation was also present (Fig. 4). It is unclear whether H_2_O_2_ accumulating in the yellow ring contributes to the resistance mechanism or reflects an early indicator of a pathway to cell death.

In addition to the *DND1* gene, silencing the *DMR6* ortholog in potato showed a significantly reduced susceptibility only at 3 days after inoculation. While, silencing the *DMR1* or *PMR4* orthologs in potato did not reduce the susceptibility to *B. cinerea*. In Arabidopsis, *dmr1* mutants showed resistance to obligate (hemi-)biotrophic oomycetes and fungal pathogens, including *Hyaloperonospora arabidopsidis, Oidium neolycopercisi, Fusarium culmorum* and *F. graminearum* [50–52]. The *DMR1* gene encodes a homoserine kinase and the resistance in the Arabidopsis *dmr1* mutants was associated with accumulation of homoserine. In pea, it has been reported that homoserine and asparagine trigger the expression of *pel*D (pectin-inducible *pel* gene D) to assist the pathogenic process of the necrotrophic root pathogen *Nectria hematococca* [53]. It is unclear whether accumulating amino acids plays a role in resistance/susceptibility to *B. cinerea*. In the *dmr1* mutants, the SA-dependent defence marker gene *PR-1* was not induced [50]. In contrast, the Arabidopsis *pmr4* mutants show constitutive activation of SA-dependent defences [54]. The *PMR4* gene encodes a callose synthase required for callose deposition in papillae and *pmr4* mutants are resistant to powdery mildews [52; 55]. There is evidence showing that SA accumulation rather than lack of pathogen-induced *PMR4* callose synthase activity is the cause of resistance in *pmr4* mutants to powdery mildews. The *DMR6* gene encodes a 2-oxoglutarate Fe (II) dependent oxygenase. It was shown that resistance to *Pseudomonas syringae* and *Phytophthora capsici* in Arabidopsis *dmr6* mutants was associated with elevated SA levels [55]. Many studies have demonstrated that SA suppresses JA signalling [56]. Since the SA signalling pathway is essential for resistance toward biotrophic organisms, while JA mounts defence against necrotrophic organisms [57], it is an intriguing question whether SA pathway play a role in reduced susceptibility only to *P. infestans* by silencing *PMR4* and *DMR6* in potato.

Our results showed that silencing the *DND1* ortholog in both potato and tomato led to reduced susceptibility to *B. cinerea* (this study) as well as powdery mildew and *P. infestans* [23]. Although further studies need to be performed to examine whether structural changes of epidermal cells and/or hormone signalling are involved in the reduced susceptibility of *DND1* well*-*silenced potato and tomato plants, our results demonstrate that knocking-down plant susceptibility genes may open a new way for breeding crops with resistance to all kind of pathogens including *B. cinerea*. The reported Arabidopsis genes (Additional file 1: Table S1) that facilitate *B. cinerea* infection offer a convenient start for a targeted homology-based approach in finding and modifying their orthologs in crops, as performed in this study. Most of these Arabidopsis genes are involved in the cell wall biogenesis and altered plant morphology is detected in their mutants (Additional file 1: Table S1), which can hamper their application in crops due to fitness costs. The Arabidopsis *dnd1* mutant showed dwarfing and auto-necrosis, and also the *DND1-*silenced potato and tomato plants were obviously affected in their development and fitness under the growth conditions used. However, fitness costs are not always associated with mutants in cell wall architecture. For example, the Arabidopsis *rwa2* mutant displayed both a normal plant morphology and resistance to *B. cinerea* [58]. Moreover, new techniques, such as CRISPR/Cas9 [59, 60] are available to create allelic loss-of-function variants, which combine favourable disease resistance levels with less detrimental side effects. With this technology it is even possible to create tetra-allelic gene disruption in tetraploid potato in one step [61].

## Conclusions

In this study, we tested whether impairing the functionality of four *S* genes, *DND1, DMR6*, *DMR1* and *PMR4*, in potato/tomato would confer resistance to *B. cinerea*. Our results showed that silencing *DND1* significantly reduced susceptibility of potato and tomato to *B. cinerea*. Microscopic studies showed that conidial germination and attachment as well as hyphal growth of *B. cinerea* on the leaf surface of DND1-silenced potato and tomato plants were hampered. Further studies need to be performed to elucidate the precise mechanisms for resistance to *B. cinerea* conferred by silencing *DND1* in plants. Our findings demonstrate that knocking-down plant susceptibility genes can open a new way for breeding crops with resistance to *B. cinerea*.

## Methods

### Plant material

pHellsgate plasmids with the *S* gene silencing fragments of *StDMR1*, *StDMR6*, *StPMR4* and *StDND1* [33] were transferred to *A. tumefaciens* strain AGL1 + virG through electroporation. RNAi transformants of potato cv Desiree for those four *S* genes were previously described [33]. Three-week old rooted in vitro plants were transplanted into soil and grown in the greenhouse at 75% relative humidity under 16-h light/ 8-h dark conditions. After 4–6 weeks, when the plants were at 9-10^th^ leaf stage, the fourth or fifth fully developed leaf (counted from the bottom) was selected for inoculation. One composite leaf with five leaflets was kept in floral foam in a plastic box covered with a transparent lid.

For *StDMR1*, *StDMR6* and *StPMR4,* two well-silenced transformants for each gene were used in a DLA. For *StDND1*, three *StDND1* well-silenced transformants (DND1A-5, DND1A-8 and DND1A-17) and one weakly-silenced transformant (DND1A-6) as well as wild type cv Desiree were used for DLA. Transformants DND1A-6, DND1A-5, DND1A-17 and cv Desiree were used for staining of fungal hyphae and analysis of defense responses. Four cuttings from each genotype were used in the analysis. Tomato *SlDND1-*silenced T2 plants were obtained by selfing primary transformants as described previously [23]. Tomato cultivar Moneymaker (MM) and wild relative *Solanum habrochaites* accession LYC4, showing reduced susceptibility to *B. cinerea* [62], were grown with *SlDND1-*silenced T2 plants in the greenhouse. The 4-6^th^ leaves were harvested from 4 to 5 week-old tomato plants and used for analysis.

### Inoculation and sample collection

The leaflets were inoculated on the adaxial side with 6–8 droplets of 2 μl of the wild type *B. cinera* strain B05.10 or a GMO reporter strain of *B. cinerea* (strain SAS56 with a pCutGUS; [34] spore suspension in PDB (Potato dextrose broth, 12 g/L) at a density of 3 × 10^5^ spores/ml. At the same time, extra plants from each genotype were mock-inoculated with only PDB medium as a control. After inoculation, leaves were incubated in closed plastic trays to obtain a humidity of 100% at 20 °C (16 h light / 8 h dark). In the DLA, the lesion diameter on leaflets was measured using a calliper with digital display (DIGI-MET^®^, Helios Preisser, Germany) at 1, 2 and 3 days post inoculation (dpi). For GUS (beta-glucuronidase) and DAB (3,3′-diaminobenzidine) staining, leaf discs (including the inoculation sites) were punched out with a 1-cm-diameter cork borer at six time points (0.5, 3, 6, 10, 24, and 48 hpi), from five leaves (three inoculated biological replicates plus two mock-inoculated controls) per time point. Leaf samples were collected for RNA extraction at each time point.

### Histological study

For histochemical localization of GUS activity, leaf discs of potato and tomato were incubated with 0.5 mg/ml X-Gluc (5-bromo-4-chloro-3-indolyl b-D glucuronide, Biosynth AG) in 50 mM phosphate buffer (pH 7.0, 1 mM KFeCN and 0.05% (*v*/v) Triton-X100) overnight at 37 °C. The staining solution was removed and leaf discs were washed with 96% ethanol until tissue was cleared. The tissue was mounted on microscope slides in 50% (v/v) glycerol.

In order to detect H_2_O_2_ generation in detached plant leaves, DAB staining was performed. Leaf discs were placed in a solution of 1 mg/mL 3,3′-diaminobenzidine dissolved in 0.2 M PBS (phosphate buffer) and HCl was used to adjust the pH to 3.8. The leaf samples were placed overnight in light to optimize the staining reaction. The samples were cleared by boiling with 96% ethanol until the leaves were decolorized and transferred to fresh 70% ethanol for storage until microscopic examination.

Twelve preparations were observed for each genotype per time point with a bright field microscope (Zeiss, Germany). Pictures were taken with a Canon PowerShot A620 camera mounted on the microscope. The presence of H_2_O_2_ is visualized as a brown coloration.

### Gene expression analysis

Leaf samples were collected at 6 time points (0.5, 3, 6, 10, 24, and 48 hpi) and stored at −80 °C. Leaf material, when taken from the −80 °C freezer, was kept in liquid nitrogen until grinding by using mortar and pestle. Total RNA was extracted by using the MagMAX-96 total RNA Isolation kit (Ambion). RNA was treated with RNAse-free DNAse (Qiagen). The concentration of isolated RNA was measured by using the Isogen Nanodrop Spectrophotometer ND-1000. cDNA was made by using the iScript cDNA synthesis kit (Bio-Rad). For the determination of relative transcript levels, the iQ SYBR Green supermix (Bio-Rad) and the C1000™ Thermal Cycler PCR system (Bio-Rad) was used. Housekeeping genes *EF1α* (plant) and actin *Bcact*A (*Botrytis*) were used for normalization. Relative transcript levels were determined using the 2^-ΔΔCt^ method [63]. Gene-specific primer pairs were designed with Primer 3.0 (http://bioinfo.ut.ee/primer3-0.4.0/primer3/, Additional file 5: Table S2). Three technical replicates were used.

## Additional files

Additional file 1: Table S1. Possible *Botrytis cinerea S* genes identified in Arabidopsis (according to Van Schie and Takken [18]). (PDF 84 kb)

Additional file 2: Figure S1. Detached leaf assay (DLA) of potato RNAi transformants with *Botrytis cinerea* (strain B05.10). **a**-**d** Lesion diameter on the inoculated leaves of the *StDMR1*, *StDMR6*, *StPMR4*, or *StDND1* well-silenced potato RNAi transformants. Two to four independent transformants per gene were used. For *StDND1*, four independent transformants were used, one weakly-silenced transformant (−), and three well-silenced transformants (+). Susceptible control was cv Desiree. One leaf (the 5^th^ or 6^th^ leaf) with 5 leaflets per plant was drop inoculated (6 to 8 drops per leaf). Data were collected at three time points: 1, 2, and 3 days post inoculation (dpi)*.* An average of all lesion diameters per transformant and per time point was calculated. (TIFF 933 kb)

Additional file 3: Figure S2. Detached leaf assay (DLA) of potato RNAi transformants with *Botrytis cinerea* (strain B05.10). Infection symptoms on cv Desiree, *StDMR1*, *StDMR6*, and *StPMR4* well-silenced transformants. One leaf (the 5^th^ or 6^th^ leaf) with 5 leaflets per plant was drop inoculated (2 to 4 drops per leaf). Photos were taken at 3 days post inoculation (dpi). (TIFF 1419 kb)

Additional file 4: Figure S3. Differences in interaction of *B. cinerea* with cv Desiree or with *StDND1-*silenced potato plants at different time points after inoculation (Scale bar = 100 μm). The photos were taken at 0.5 h post inoculation (hpi), 3 hpi, 6 hpi, 10 hpi and 24 hpi, respectively. (TIFF 4981 kb)

Additional file 5: Table S2. Primers used in this study. (PDF 143 kb)

## Abbreviations

ABA: abscisic acid
BDG: bodyguard
CNGC: cyclic nucleotide-gated ion channel
CRISPR/Cas9: Clustered regularly interspaced short palindromic repeats/ CRISPR-associated protein-9 nuclease
cv: cultivar
DAB: 3,3′-diaminobenzidine
DLA: detached leaf assay
DND: defence no death
dpi: days post inoculation
endo-PGs: endopolygalacturonases
GUS: beta-glucuronidase
hpi: hour post inoculation
HR: hypersensitive response
LACS: long-chain acyl-CoA synthetase
LCR: lacerate
MM: Moneymaker
PBS: phosphate buffer
PDB: Potato dextrose broth
PMEs: pectin methylesterases
QTLs: quantitative trait loci
RST: resurrection
*S*: susceptibility
T2: the second generation
X-Gluc: 5-bromo-4-chloro-3-indolyl b-D glucuronide

## References

[1] Dean R, Van Kan JA, Pretorius ZA, Hammond-Kosack KE, Di Pietro A, Spanu PD, Rudd JJ, Dickman M, Kahmann R, Ellis J (2012). The top 10 fungal pathogens in molecular plant pathology. Mol Plant Pathol.

[2] van Kan JA (2006). Licensed to kill: the lifestyle of a necrotrophic plant pathogen. Trends Plant Sci.

[3] Brito N, Espino JJ, González C (2006). The endo-β-1, 4-xylanase Xyn11A is required for virulence in *Botrytis cinerea*. Mol Plant-Microbe Interact.

[4] Kars I, van Kan JA: Extracellular enzymes and metabolites involved in pathogenesis of botrytis. In: Botrytis: Biology, pathology and control. Springer; 2007: 99–118.

[5] McKeen W (1974). Mode of penetration of epidermal cell walls of Vicia Faba by *Botrytis cinerea*. Phytopathology.

[6] Noda J, Brito N, González C (2010). The Botrytis Cinerea xylanase Xyn11A contributes to virulence with its necrotizing activity, not with its catalytic activity. BMC Plant Biol.

[7] Doss RP, Potter SW, Chastagner GA, Christian JK, Fukunaga LE (1993). Adhesion of nongerminated *Botrytis cinerea* conidia to several substrata. Appl Environ Microbiol.

[8] Doss RP, Potter SW, Soeldner AH, Christian JK, Fukunaga LE (1995). Adhesion of germlings of *Botrytis cinerea*. Appl Environ Microbiol.

[9] Prins TW, Tudzynski P, Von Tiedemann A, Tudzysnki B, ten Have A, Hansen ME, Tenberge K, van Kan JAL (2000). Infection strategies of *Botrytis cinerea* and related necrotrophic pathogens. *Fungal Pathology*. Kluwer academic publishers.

[10] Gil-ad NL, Bar-Nun N, Mayer AM (2001). The possible function of the glucan sheath of *Botrytis cinerea*: effects on the distribution of enzyme activities. FEMS Microbiol Lett.

[11] Gourgues M, Brunet-Simon A, Lebrun MH, Levis C (2004). The tetraspanin BcPls1 is required for appressorium-mediated penetration of *Botrytis cinerea* into host plant leaves. Mol Microbiol.

[12] Benito EP, ten Have A, Van 't Klooster JW, JAL v K (1998). Fungal and plant gene expression during synchronized infection of tomato leaves by *Botrytis cinerea*. Eur J Plant Pathol.

[13] Tenberge KB Morphology and cellular organisation in *Botrytis* interactions with plants. In: *Botrytis: Biology, pathology and control*. Kluwer academic publishers; 2007:67–84.

[14] Denby KJ, Kumar P, Kliebenstein DJ (2004). Identification of *Botrytis cinerea* susceptibility loci in *Arabidopsis thaliana*. Plant J.

[15] Finkers R, van Heusden AW, Meijer-Dekens F, van Kan JA, Maris P, Lindhout P (2007). The construction of a *Solanum habrochaites* LYC4 introgression line population and the identification of QTLs for resistance to *Botrytis cinerea*. Theor Appl Genet.

[16] Anuradha C, Gaur PM, Pande S, Gali KK, Ganesh M, Kumar J, Varshney RK, Mapping QTL (2011). For resistance to botrytis grey mould in chickpea. Euphytica.

[17] Zhang W, Kwon S-T, Chen F, Kliebenstein DJ. Isolate dependency of *Brassica rapa* resistance QTLs to *Botrytis cinerea*. Front Plant Sci. 2016;7 10.3389/fpls.2016.00161 PMC4756292 26925079

[18] van Schie CC, Takken FL (2014). Susceptibility genes 101: how to be a good host. Annu Rev Phytopathol.

[19] Cantu D, Vicente A, Greve L, Dewey F, Bennett A, Labavitch J, Powell A (2008). The intersection between cell wall disassembly, ripening, and fruit susceptibility to *Botrytis cinerea*. Proc Natl Acad Sci.

[20] Hernández-Blanco C, Feng DX, Hu J, Sánchez-Vallet A, Deslandes L, Llorente F, Berrocal-Lobo M, Keller H, Barlet X, Sánchez-Rodríguez C (2007). Impairment of cellulose synthases required for Arabidopsis secondary cell wall formation enhances disease resistance. Plant Cell.

[21] Bai Y, Pavan S, Zheng Z, Zappel NF, Reinstädler A, Lotti C, De Giovanni C, Ricciardi L, Lindhout P, Visser R (2008). Naturally occurring broad-spectrum powdery mildew resistance in a central American tomato accession is caused by loss of mlo function. Mol Plant-Microbe Interact.

[22] Pavan S, Jacobsen E, Visser RG, Bai Y (2010). Loss of susceptibility as a novel breeding strategy for durable and broad-spectrum resistance. Mol Breed.

[23] Sun K, A-MA W, Loonen AE, Huibers RP, van der Vlugt R, Goverse A, Jacobsen E, Visser RG, Bai Y (2016). Down-regulation of Arabidopsis DND1 orthologs in potato and tomato leads to broad-spectrum resistance to late blight and powdery mildew. Transgenic Res.

[24] Van Damme M, Andel A, Huibers RP, Panstruga R, Weisbeek PJ, Van den Ackerveken G (2005). Identification of Arabidopsis loci required for susceptibility to the downy mildew pathogen *Hyaloperonospora parasitica*. Mol Plant-Microbe Interact.

[25] Vogel J, Somerville S (2000). Isolation and characterization of powdery mildew-resistant Arabidopsis mutants. Proc Natl Acad Sci.

[26] Audenaert K, De Meyer GB, Höfte MM (2002). Abscisic acid determines basal susceptibility of tomato to *Botrytis cinerea* and suppresses salicylic acid-dependent signaling mechanisms. Plant Physiol.

[27] Curvers K, Seifi H, Mouille G, De Rycke R, Asselbergh B, Van Hecke A, Vanderschaeghe D, Höfte H, Callewaert N, Van Breusegem F (2010). Abscisic acid deficiency causes changes in cuticle permeability and pectin composition that influence tomato resistance to *Botrytis cinerea*. Plant Physiol.

[28] Asselbergh B, Curvers K, França SC, Audenaert K, Vuylsteke M, Van Breusegem F, Höfte M (2007). Resistance to *Botrytis cinerea* in sitiens, an abscisic acid-deficient tomato mutant, involves timely production of hydrogen peroxide and cell wall modifications in the epidermis. Plant Physiol.

[29] Bessire M, Chassot C, Jacquat AC, Humphry M, Borel S, Petétot JMC, Métraux JP, Nawrath C (2007). A permeable cuticle in Arabidopsis leads to a strong resistance to *Botrytis cinerea*. EMBO J.

[30] Genger RK, Jurkowski GI, McDowell JM, Lu H, Jung HW, Greenberg JT, Bent AF (2008). Signaling pathways that regulate the enhanced disease resistance of Arabidopsis “defense, no death” mutants. Mol Plant-Microbe Interact.

[31] Jurkowski GI, Smith Jr RK, Yu I-c, Ham JH, Sharma SB, Klessig DF, Fengler KA, Bent AF (2004). Arabidopsis DND2, a second cyclic nucleotide-gated ion channel gene for which mutation causes the “defense, no death” phenotype. Mol Plant-Microbe Interact.

[32] Yu I-c, Fengler KA, Clough SJ, Bent AF (2000). Identification of Arabidopsis mutants exhibiting an altered hypersensitive response in gene-for-gene disease resistance. Mol Plant-Microbe Interact.

[33] Sun K, Wolters A-MA, Vossen JH, Rouwet ME, Loonen AE, Jacobsen E, Visser RG, Bai Y (2016). Silencing of six susceptibility genes results in potato late blight resistance. Transgenic Res.

[34] Van Kan J, Van't Klooster J, Wagemakers C, Dees D, van der Vlugt-Bergmans C (1997). Cutinase a of *Botrytis cinerea* is expressed, but not essential, during penetration of gerbera and tomato. Mol Plant-Microbe Interact.

[35] Liu S, Oeljeklaus S, Gerhardt B, Tudzynski B (1998). Purification and characterization of glucose oxidase of *Botrytis cinerea*. *Physiological*. Mol Plant Pathol.

[36] Rolke Y, Liu SJ, Quidde T, Williamson B, Schouten A, Weltring KM, Siewers V, Tenberge KB, Tudzynski B, Tudzynski P (2004). Functional analysis of H_2_O_2_-generating systems in *Botrytis cinerea*: the major cu-Zn-superoxide dismutase (BCSOD1) contributes to virulence on French bean, whereas a glucose oxidase (BCGOD1) is dispensable. Mol Plant Pathol.

[37] Schouten A, Tenberge KB, Vermeer J, Stewart J, Wagemakers CAM, Williamson B, van Kan JAL (2002). Functional analysis of an extracellular catalase of *Botrytis cinerea*. Mol Plant Pathol.

[38] Temme N, Tudzynski P (2009). Does Botrytis Cinerea ignore H_2_O_2_-induced oxidative stress during infection? Characterization of *Botrytis* activator protein 1. Mol Plant-Microbe Interact.

[39] Clough SJ, Fengler KA, Yu I-c, Lippok B, Smith RK, Bent AF (2000). The Arabidopsis dnd1 “defense, no death” gene encodes a mutated cyclic nucleotide-gated ion channel. Proc Natl Acad Sci.

[40] Sherman T, Fromm H: Physiological roles of cyclic nucleotide gated channels in plants. In: *Signaling in Plants.* Springer; 2009: 91–106.

[41] Govrin EM, Levine A (2000). The hypersensitive response facilitates plant infection by the necrotrophic pathogen *Botrytis cinerea*. Curr Biol.

[42] Yu I-c, Parker J, Bent AF (1998). Gene-for-gene disease resistance without the hypersensitive response in Arabidopsis dnd1 mutant. Proc Natl Acad Sci.

[43] Nawrath C (2006). Unraveling the complex network of cuticular structure and function. Curr Opin Plant Biol.

[44] Tang D, Simonich MT, Innes RW (2007). Mutations in LACS2, a long-chain acyl-coenzyme a synthetase, enhance susceptibility to avirulent *Pseudomonas syringae* but confer resistance to *Botrytis cinerea* in Arabidopsis. Plant Physiol.

[45] Chassot C, Nawrath C, Métraux JP (2007). Cuticular defects lead to full immunity to a major plant pathogen. Plant J.

[46] Mang HG, Laluk KA, Parsons EP, Kosma DK, Cooper BR, Park HC, AbuQamar S, Boccongelli C, Miyazaki S, Consiglio F (2009). The Arabidopsis RESURRECTION1 gene regulates a novel antagonistic interaction in plant defense to biotrophs and necrotrophs. Plant Physiol.

[47] Ramírez V, Agorio A, Coego A, García-Andrade J, Hernández MJ, Balaguer B, Ouwerkerk PB, Zarra I, Vera P (2011). MYB46 modulates disease susceptibility to *Botrytis cinerea* in Arabidopsis. Plant Physiol.

[48] Ramírez V, García-Andrade J, Vera P (2011). Enhanced disease resistance to *Botrytis cinerea* in myb46 Arabidopsis plants is associated to an early down-regulation of CesA genes. Plant Signal Behav.

[49] Ferrari S, Plotnikova JM, De Lorenzo G, Ausubel FM (2003). Arabidopsis local resistance to *Botrytis cinerea* involves salicylic acid and camalexin and requires EDS4 and PAD2, but not SID2, EDS5 or PAD4. Plant J.

[50] Van Damme M, Zeilmaker T, Elberse J, Andel A, De Sain-van d, Velden M, Van den Ackerveken G (2009). Downy mildew resistance in Arabidopsis by mutation of HOMOSERINEKINASE. Plant Cell.

[51] Huibers RP, Loonen AEHM, Gao D, Van den Ackerveken G, Richard GF, Visser RGF, Bai Y (2013). Powdery mildew resistance in tomato by impairment of SlPMR4 and SlDMR1. PLoS One.

[52] Brewer HC, Hawkins ND, Hammond-Kosack KE (2014). Mutations in the Arabidopsis homoserine kinase gene *DMR1* confer enhanced resistance to *Fusarium culmorum* and *F. graminearum*. BMC Plant Biol.

[53] Yang A, Rogers LM, Song Y, Guo W, Kolattukudy PE (2005). Homoserine and asparagine are host signals that trigger in planta expression of a pathogenesis gene in *Nectria haematococca*. Proc Natl Acad Sci.

[54] Nishimura MT, Stein M, Hou B-H, Vogel JP, Edwards H, Somerville S (2003). Loss of a callose synthase results in salicylic acid–dependent disease resistance. Science.

[55] Zeilmaker T, Ludwig NR, Elberse J, Seidl MF, Berke L, Van Doorn A, Robert C, Schuurink RC, Snel B, Van den Ackerveken G, RESISTANT DOWNYMILDEW (2015). 6 and DMR6-LIKE OXYGENASE 1 are partially redundant but distinct suppressors of immunity in Arabidopsis. Plant J.

[56] Van d, Does D, Leon-Reyes A, Koornneef A, van Verk MC, Rodenburg N, Pauwels L, Goossens A, Körbes AP, Memelink J, Ritsema T, SCM VW, CMJ P (2013). Salicylic acid suppresses Jasmonic acid signaling downstream of SCFCOI1-JAZ by targeting GCC promoter motifs via transcription factor ORA59. Plant Cell.

[57] Robert-Seilaniantz A, Grant M, Jones JDG (2011). Hormone crosstalk in plant disease and defense: more than just JASMONATE-SALICYLATE antagonism. Annu Rev Phytopathol.

[58] Manabe Y, Nafisi M, Verhertbruggen Y, Orfila C, Gille S, Rautengarten C, Cherk C, Marcus SE, Somerville S, Pauly M (2011). Loss-of-function mutation of REDUCED WALL ACETYLATION2 in Arabidopsis leads to reduced cell wall ACETYLATION and increased resistance to *Botrytis cinerea*. Plant Physiol.

[59] Belhaj K, Chaparro-Garcia A, Kamoun S, Nekrasov V (2013). Plant genome editing made easy: targeted mutagenesis in model and crop plants using the CRISPR/Cas system. Plant Methods.

[60] Xie K, Yang Y (2013). RNA-guided genome editing in plants using a CRISPR–Cas system. Mol Plant.

[61] Andersson M, Turesson H, Nicolia A, Fält A-S, Samuelsson M, Hofvander P (2017). Efficient targeted multiallelic mutagenesis in tetraploid potato (Solanum Tuberosum) by transient CRISPR-Cas9 expression in protoplasts. Plant Cell Rep.

[62] Ten Have A, van Berloo R, Lindhout P, van Kan JA (2007). Partial stem and leaf resistance against the fungal pathogen Botrytis Cinerea in wild relatives of tomato. Eur J Plant Pathol.

[63] Livak KJ, Schmittgen TD (2001). Analysis of relative gene expression data using real-time quantitative PCR and the 2^− ΔΔCT^ method. methods.

