# Supplementary material for: Silencing of DND1 in potato and tomato impedes conidial germination, attachment and hyphal growth of Botrytis cinerea
Source: BMC Plant Biol. 2017 Dec 6;17:235. doi: 10.1186/s12870-017-1184-2 (PMC5719932; doi:10.1186/s12870-017-1184-2)
Supplement: Supplementary file 1 — Possible Botrytis cinerea S genes identified in Arabidopsis (according to Van Schie and Takken [18]). (PDF 84 kb) [file 12870_2017_1184_MOESM1_ESM.pdf]

Table S1. Possible *Botrytis cinerea S* genes identified in Arabidopsis (according to van Schie and Takken [1])

Gene	Plant species	Susceptibility mechanism	Reported pleiotropic phenotype	References
LePG and *LeEXP1*	Tomato	Cell wall architecture of fruit affects penetration and colonization of *Botrytis*	Reduced fruit softening	[2]
LACS2/ SMA4/ BRE1	Arabidopsis	Cuticle permeability might affect diffusion of elicitors, ROS and defense compounds	Permeable cuticle, subtle leaf deformation and organ fusion, more susceptible to *Pseudomonas*, increased sensitivity to salt and drought	[3], [4]
RWA2	Arabidopsis	Pathogen entry? Unknown, Cuticle/wall permeability affects diffusion of elicitors, ROS and defense compounds?	None reported	[5]
FDH	Arabidopsis	Cuticle permeability might affect diffusion of elicitors, ROS and defense compounds	Some organ fusion	[6]

LCR	Arabidopsis	Cuticle permeability might affect diffusion of elicitors, ROS and defense compounds	Some organ fusion	[6], [7]
ATT1	Arabidopsis	Cuticle permeability might affect diffusion of elicitors, ROS and defense compounds	Permeable cuticle, increased susceptibility to *Pseudomonas*	[4], [8]
BDG	Arabidopsis	Cuticle permeability might affect diffusion of elicitors, ROS and defense compounds	Permeable cuticle, more sensitive to osmotic stress, narrow elongated leaves, smaller plants	[6, 9-11]
Sit	Tomato	ABA affects cuticle composition and defense. Mutant has decreased ABA and increased cuticle permeability (and DAMP induced defense?)	Increased sensitivity to drought, wilting (open stomata), impaired interaction of (beneficial) arbuscular mycorrhizal fungi, early germination (e.g. viviparous)	[11-17]
CESA4/7	Arabidopsis	Mutant has increased ABA/JA/Eth	None reported	[18]
CESA8	Arabidopsis	Mutant has increased ABA/JA/Eth	Increased ABA, enhanced drought tolerance	[18, 19]

MYB46	Arabidopsis	Regulates CESA4/7/8, CSLA9; probable DAMP induced defense suppression	None reported	[20-22]
DND1/2	Arabidopsis	Defense suppression, HR and positive regulator of NO synthesis	Smaller plant, early senescence, moderate lesion mimic	[23-28]
SR1/ CAMTA3	Arabidopsis	Defense suppression SA	Smaller plants [abolished at higher temp], increased susceptibility to insect feeding (*Trichoplusia*, *Bradysia*), possible reduced cold tolerance	[20, 29-34]
RST1	Arabidopsis	Defense suppression JA; mutant has increased free (cuticular) lipids and increased JA	Increased sensitivity to biotroph powdery mildew (E. cichoracearum), increased embryo abortion, reduced storage lipids in seed	[19, 35]
PLP2	Arabidopsis	Defense suppression, cell death (oxylipin mediated)	Increased sensitivity to virus (Cucumber mosaic virus), no developmental phenotype reported	[36, 37]

References

1. van Schie CC, Takken FL: Susceptibility genes 101: how to be a good host. *Annual review of phytopathology* 2014, 52:551-581.
2. Cantu D, Vicente A, Greve L, Dewey F, Bennett A, Labavitch J, Powell A: The intersection between cell wall disassembly, ripening, and fruit susceptibility to *Botrytis cinerea*. *Proceedings of the National Academy of Sciences* 2008, 105(3):859-864.
3. Bessire M, Chassot C, Jacquat AC, Humphry M, Borel S, Petétot JMC, Métraux JP, Nawrath C: A permeable cuticle in Arabidopsis leads to a strong resistance to *Botrytis cinerea*. *The EMBO Journal* 2007, 26(8):2158-2168.
4. Tang D, Simonich MT, Innes RW: Mutations in LACS2, a long-chain acyl-coenzyme A synthetase, enhance susceptibility to avirulent *Pseudomonas syringae* but confer resistance to *Botrytis cinerea* in Arabidopsis. *Plant Physiology* 2007, 144(2):1093-1103.
5. Manabe Y, Nafisi M, Verhertbruggen Y, Orfila C, Gille S, Rautengarten C, Cherk C, Marcus SE, Somerville S, Pauly M: Loss-of-function mutation of REDUCED WALL ACETYLATION2 in Arabidopsis leads to reduced cell wall acetylation and increased resistance to *Botrytis cinerea*. *Plant physiology* 2011, 155(3):1068-1078.
6. Voisin D, Nawrath C, Kurdyukov S, Franke RB, Reina-Pinto JJ, Efremova N, Will I, Schreiber L, Yephremov A: Dissection of the complex phenotype in cuticular mutants of Arabidopsis reveals a role of SERRATE as a mediator. 2009.
7. Wellesen K, Durst F, Pinot F, Benveniste I, Nettesheim K, Wisman E, Steiner-Lange S, Saedler H, Yephremov A: Functional analysis of the LACERATA gene of Arabidopsis provides evidence for different roles of fatty acid ω-hydroxylation in development. *Proceedings of the National Academy of Sciences* 2001, 98(17):9694-9699.

8. Xiao F, Goodwin SM, Xiao Y, Sun Z, Baker D, Tang X, Jenks MA, Zhou JM: Arabidopsis CYP86A2 represses *Pseudomonas syringae* type III genes and is required for cuticle development. *The EMBO journal* 2004, 23(14):2903-2913.
9. Kurdyukov S, Faust A, Nawrath C, Bär S, Voisin D, Efremova N, Franke R, Schreiber L, Saedler H, Métraux J-P: The epidermis-specific extracellular BODYGUARD controls cuticle development and morphogenesis in Arabidopsis. *The Plant Cell* 2006, 18(2):321-339.
10. Wang D, Dong X: A highway for war and peace: the secretory pathway in plant–microbe interactions. *Molecular plant* 2011, 4(4):581-587.
11. Asselbergh B, Achuo AE, Höfte M, Van Gijsegem F: Abscisic acid deficiency leads to rapid activation of tomato defence responses upon infection with *Erwinia chrysanthemi*. *Molecular Plant Pathology* 2008, 9(1):11-24.
12. Curvers K, Seifi H, Mouille G, De Rycke R, Asselbergh B, Van Hecke A, Vanderschaeghe D, Höfte H, Callewaert N, Van Breusegem F: Abscisic acid deficiency causes changes in cuticle permeability and pectin composition that influence tomato resistance to *Botrytis cinerea*. *Plant Physiology* 2010, 154(2):847-860.
13. Groot SP, Karssen CM: Dormancy and germination of abscisic acid-deficient tomato seeds Studies with the sitiens mutant. *Plant Physiology* 1992, 99(3):952-958.
14. Harrison E, Burbidge A, Okyere J, Thompson AJ, Taylor IB: Identification of the tomato ABA-deficient mutant sitiens as a member of the ABA-aldehyde oxidase gene family using genetic and genomic analysis. *Plant Growth Regulation* 2011, 64(3):301-309.
15. Herrera-Medina MJ, Steinkellner S, Vierheilig H, Ocampo Bote JA, García Garrido JM: Abscisic acid determines arbuscule development and functionality in the tomato arbuscular mycorrhiza. *New Phytologist* 2007, 175(3):554-564.

16. Tal M: Abnormal stomatal behavior in wilty mutants of tomato. *Plant Physiology* 1966, 41(8):1387-1391.

17. Audenaert K, De Meyer GB, Höfte MM: Abscisic acid determines basal susceptibility of tomato toBotrytis cinerea and suppresses salicylic acid-dependent signaling mechanisms. *Plant Physiology* 2002, 128(2):491-501.

18. Hernández-Blanco C, Feng DX, Hu J, Sánchez-Vallet A, Deslandes L, Llorente F, Berrocal-Lobo M, Keller H, Barlet X, Sánchez-Rodríguez C: Impairment of cellulose synthases required for Arabidopsis secondary cell wall formation enhances disease resistance. *The Plant Cell* 2007, 19(3):890-903.

19. Chen X, Goodwin SM, Liu X, Chen X, Bressan RA, Jenks MA: Mutation of the RESURRECTION1 locus of Arabidopsis reveals an association of cuticular wax with embryo development. *Plant physiology* 2005, 139(2):909-919.

20. Kim WC, Ko JH, Kim JY, Kim J, Bae HJ, Han KH: MYB46 directly regulates the gene expression of secondary wall-associated cellulose synthases in Arabidopsis. *The Plant Journal* 2013, 73(1):26-36.

21. Ramírez V, Agorio A, Coego A, García-Andrade J, Hernández MJ, Balaguer B, Ouwerkerk PB, Zarra I, Vera P: MYB46 modulates disease susceptibility to *Botrytis cinerea* in Arabidopsis. *Plant Physiology* 2011, 155(4):1920-1935.

22. Ramírez V, García-Andrade J, Vera P: Enhanced disease resistance to *Botrytis cinerea* in myb46 Arabidopsis plants is associated to an early down-regulation of CesA genes. *Plant signaling & behavior* 2011, 6(6):911-913.

23. Ahn IP: Disturbance of the Ca^{2+}/calmodulin-dependent signalling pathway is responsible for the resistance of Arabidopsis dnd1 against *Pectobacterium carotovorum* infection. *Molecular plant pathology* 2007, 8(6):747-759.

24. Clough SJ, Fengler KA, Yu I-c, Lippok B, Smith RK, Bent AF: The Arabidopsis dnd1 “defense, no death” gene encodes a mutated cyclic nucleotide-gated ion channel. *Proceedings of the National Academy of Sciences* 2000, 97(16):9323-9328.

25. Genger RK, Jurkowski GI, McDowell JM, Lu H, Jung HW, Greenberg JT, Bent AF: Signaling pathways that regulate the enhanced disease resistance of Arabidopsis “defense, no death” mutants. *Molecular Plant-Microbe Interactions* 2008, 21(10):1285-1296.

26. Govrin EM, Levine A: The hypersensitive response facilitates plant infection by the necrotrophic pathogen *Botrytis cinerea*. *Current biology* 2000, 10(13):751-757.

27. Jurkowski GI, Smith Jr RK, Yu I-c, Ham JH, Sharma SB, Klessig DF, Fengler KA, Bent AF: Arabidopsis DND2, a second cyclic nucleotide-gated ion channel gene for which mutation causes the “defense, no death” phenotype. *Molecular plant-microbe interactions* 2004, 17(5):511-520.

28. Su’udi M, Kim MG, Park S-R, Hwang D-J, Bae S-C, Ahn I-P: Arabidopsis cell death in compatible and incompatible interactions with *Alternaria brassicicola*. *Molecules and cells* 2011, 31(6):593-601.

29. Doherty CJ, Van Buskirk HA, Myers SJ, Thomashow MF: Roles for Arabidopsis CAMTA transcription factors in cold-regulated gene expression and freezing tolerance. *The Plant Cell* 2009, 21(3):972-984.

30. Du L, Ali GS, Simons KA, Hou J, Yang T, Reddy A, Poovaiah B: Ca^{2+}/calmodulin regulates salicylic-acid-mediated plant immunity. *Nature* 2009, 457(7233):1154-1158.

31. Galon Y, Nave R, Boyce JM, Nachmias D, Knight MR, Fromm H: Calmodulin-binding transcription activator (CAMTA) 3 mediates biotic defense responses in Arabidopsis. *FEBS letters* 2008, 582(6):943-948.

32. Laluk K, Prasad K, Savchenko T, Celesnik H, Dehesh K, Levy M, Mitchell-Olds T, Reddy A: The calmodulin-binding transcription factor SIGNAL RESPONSIVE1 is a novel regulator of glucosinolate metabolism and herbivory tolerance in Arabidopsis. *Plant and Cell Physiology* 2012, 53(12):2008-2015.

33. Nie H, Zhao C, Wu G, Wu Y, Chen Y, Tang D: SR1, a calmodulin-binding transcription factor, modulates plant defense and ethylene-induced senescence by directly regulating NDR1 and EIN3. *Plant physiology* 2012, 158(4):1847-1859.

34. Qiu Y, Xi J, Du L, Suttle JC, Poovaiah B: Coupling calcium/calmodulin-mediated signaling and herbivore-induced plant response through calmodulin-binding transcription factor AtSR1/CAMTA3. *Plant molecular biology* 2012, 79(1-2):89-99.

35. Mang HG, Laluk KA, Parsons EP, Kosma DK, Cooper BR, Park HC, AbuQamar S, Boccongelli C, Miyazaki S, Consiglio F: The Arabidopsis RESURRECTION1 gene regulates a novel antagonistic interaction in plant defense to biotrophs and necrotrophs. *Plant physiology* 2009, 151(1):290-305.

36. Camera SL, Balagué C, Göbel C, Geoffroy P, Legrand M, Feussner I, Roby D, Heitz T: The Arabidopsis patatin-like protein 2 (PLP2) plays an essential role in cell death execution and differentially affects biosynthesis of oxylipins and resistance to pathogens. *Molecular plant-microbe interactions* 2009, 22(4):469-481.

37. La Camera S, Geoffroy P, Samaha H, Ndiaye A, Rahim G, Legrand M, Heitz T: A pathogen-inducible patatin-like lipid acyl hydrolase facilitates fungal and bacterial host colonization in Arabidopsis. *The Plant Journal* 2005, 44(5):810-825.
