# Supplementary figures and images for: Silencing of DND1 in potato and tomato impedes conidial germination, attachment and hyphal growth of Botrytis cinerea
Source: BMC Plant Biol. 2017 Dec 6;17:235. doi: 10.1186/s12870-017-1184-2 (PMC5719932; doi:10.1186/s12870-017-1184-2)

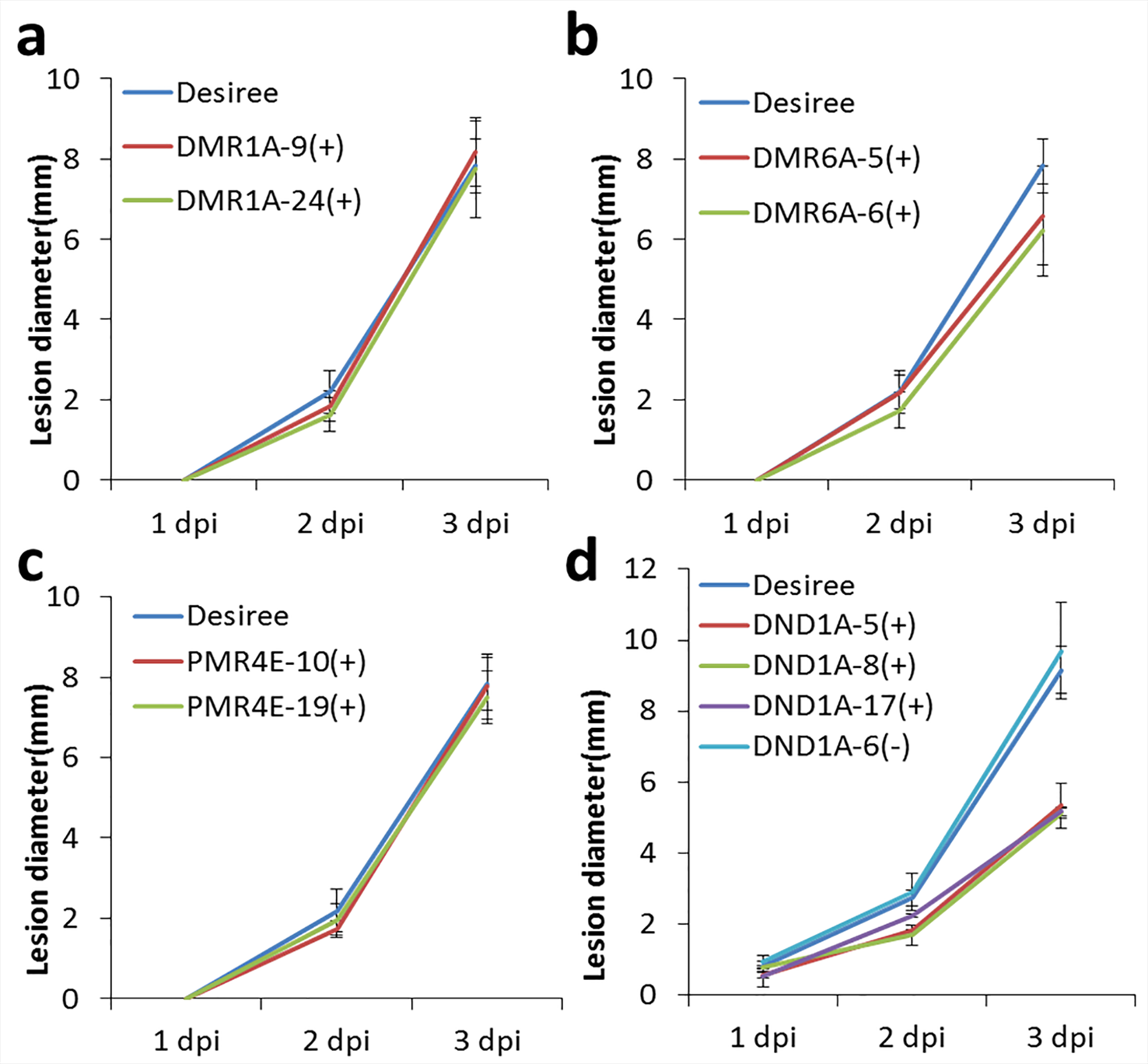

Supplement: Supplementary file 2 — Detached leaf assay (DLA) of potato RNAi transformants with Botrytis cinerea (strain B05.10). a-d Lesion diameter on the inoculated leaves of the StDMR1, StDMR6, StPMR4, or StDND1 well-silenced potato RNAi transformants. Two to four independent transformants per gene were used. For StDND1, four independent transformants were used, one weakly-silenced transformant (−), and three well-silenced transformants (+). Susceptible control was cv Desiree. One leaf (the 5th or 6th leaf) with 5 leaflets per plant was drop inoculated (6 to 8 drops per leaf). Data were collected at three time points: 1, 2, and 3 days post inoculation (dpi). An average of all lesion diameters per transformant and per time point was calculated. (TIFF 933 kb) [file 12870_2017_1184_MOESM2_ESM.tif]

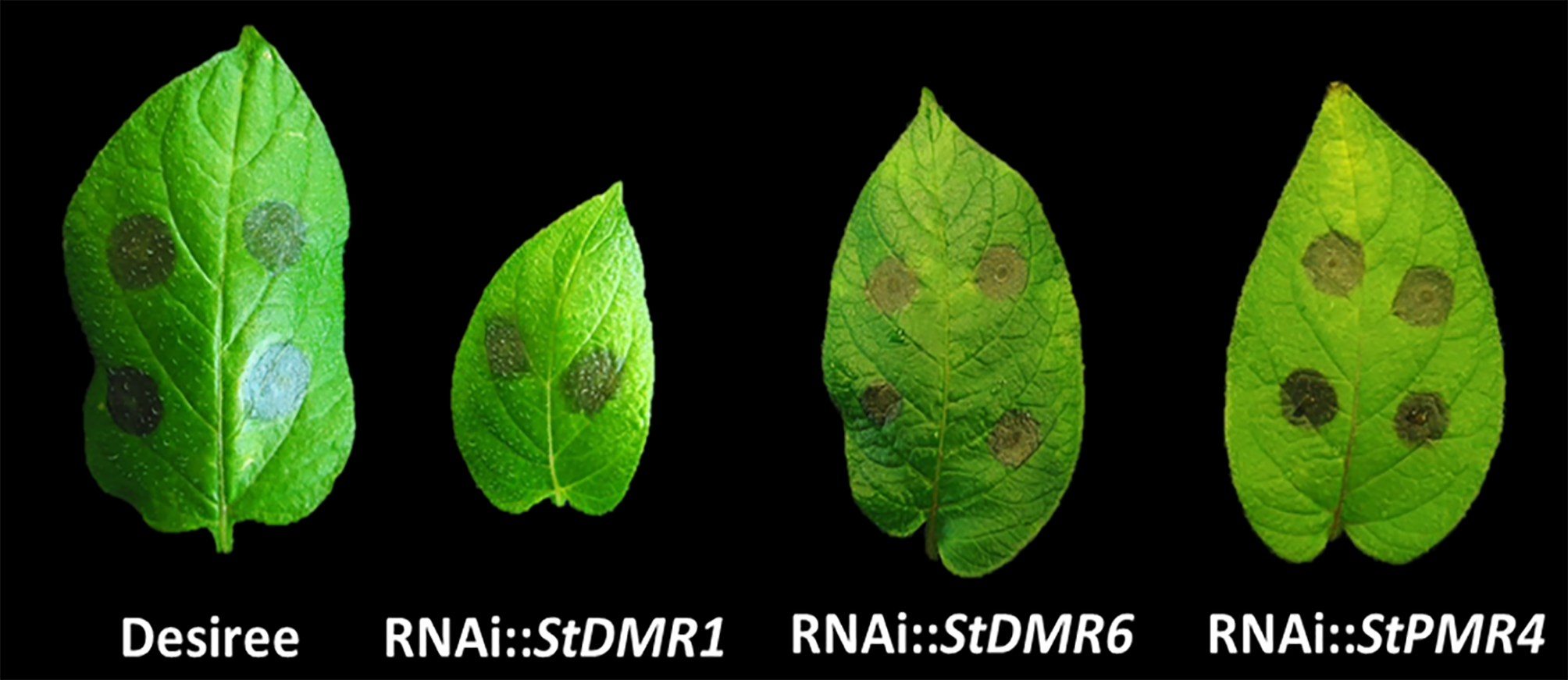

Supplement: Supplementary file 3 — Detached leaf assay (DLA) of potato RNAi transformants with Botrytis cinerea (strain B05.10). Infection symptoms on cv Desiree, StDMR1, StDMR6, and StPMR4 well-silenced transformants. One leaf (the 5th or 6th leaf) with 5 leaflets per plant was drop inoculated (2 to 4 drops per leaf). Photos were taken at 3 days post inoculation (dpi). (TIFF 1419 kb) [file 12870_2017_1184_MOESM3_ESM.tif]

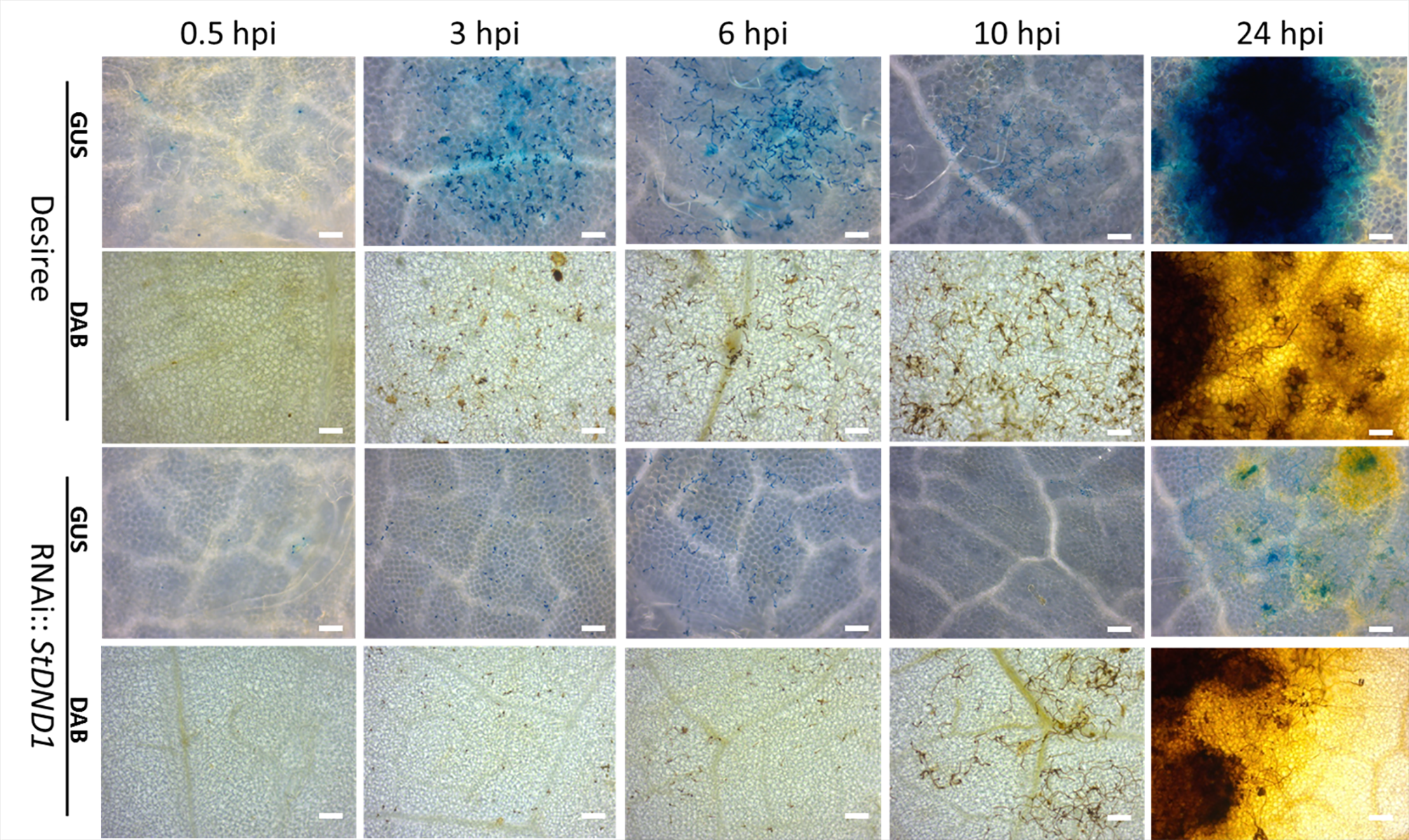

Supplement: Supplementary file 4 — Differences in interaction of B. cinerea with cv Desiree or with StDND1-silenced potato plants at different time points after inoculation (Scale bar = 100 μm). The photos were taken at 0.5 h post inoculation (hpi), 3 hpi, 6 hpi, 10 hpi and 24 hpi, respectively. (TIFF 4981 kb) [file 12870_2017_1184_MOESM4_ESM.tif]
