# Supplementary material for: Silencing of DND1 in potato and tomato impedes conidial germination, attachment and hyphal growth of Botrytis cinerea
Source: BMC Plant Biol. 2017 Dec 6;17:235. doi: 10.1186/s12870-017-1184-2 (PMC5719932; doi:10.1186/s12870-017-1184-2)
Supplement: Supplementary file 5 — Primers used in this study. (PDF 143 kb) [file 12870_2017_1184_MOESM5_ESM.pdf]

Table S2. Primers used in this study

Primer name	Sequence (5'-3')	Used for
Fw-Botrytis Actin	TCTGTCTTGGGTCTTGAGAG	*Botrytis cinerea* normalisation
Rv-Botrytis Actin	GGTGCAAGAGCAGTGATTTC	
Fw-Bcpg1	AAC GTG GTA CCG CCT GTA CC	determining relative transcript levels
Rv-Bcpg1	AGC CTT GGA CTT GGA AGC G	
Fw-BccutA	CCTCCTTCCTCTCTCCGTCT	
Rv-BccutA	GCTGGGTAGTCGACACCATT	
